# Neural basis of self-initiative in relation to apathy in a student sample

**DOI:** 10.1038/s41598-017-03564-5

**Published:** 2017-06-12

**Authors:** Claire Kos, Nicky G. Klaasen, Jan-Bernard C. Marsman, Esther M. Opmeer, Henderikus Knegtering, André Aleman, Marie-José van Tol

**Affiliations:** 1 Department of Neuroscience, section Cognitive Neuropsychiatry, University Medical Center Groningen, University of Groningen, Antonius Deusinglaan 2, 9713 AW Groningen, The Netherlands; 2 Lentis Research, Lentis Center for Mental Health Care, Hereweg 80, 9725 AG Groningen, The Netherlands; 3 Rob Giel Research Center, University Medical Center Groningen, University of Groningen, Hanzeplein 1, 9713 GZ Groningen, The Netherlands; 4 Department of Psychology, University of Groningen, Grote Kruisstraat 2/1, 9712 TS Groningen, The Netherlands

## Abstract

Human behaviour can be externally driven, e.g. catching a falling glass, or self-initiated and goal-directed, e.g. drinking a cup of coffee when one deems it is time for a break. Apathy refers to a reduction of self-initiated goal-directed or motivated behaviour, frequently present in neurological and psychiatric disorders. The amount of undertaken goal-directed behaviour varies considerably in clinical as well as healthy populations. In the present study, we investigated behavioural and neural correlates of self-initiated action in a student sample (N = 39) with minimal to high levels of apathy. We replicated activation of fronto-parieto-striatal regions during self-initiation. The neural correlates of self-initiated action did not explain varying levels of apathy in our sample, neither when mass-univariate analysis was used, nor when multivariate patterns of brain activation were considered. Other hypotheses, e.g. regarding a putative role of deficits in reward anticipation, effort expenditure or executive difficulties, deserve investigation in future studies.

## Introduction

Intentional behaviour is of critical importance for normal daily functioning. It comprises multiple components, including deciding whether to act, what action to perform, and when to execute it^1^. A disruption in intentional behaviour could lead to behavioural poverty known as apathy^2^. Apathy, i.e. a quantifiable reduction in goal-directed behaviour, is frequently present in a variety of neurological and psychiatric disorders^3^, but is also present to a certain degree in a portion of the healthy population^4–8^. In the general population apathy has been associated with lower behavioural activation, reduced perceived quality of life^6^, and higher levels of distress^8^, similar to the clinical manifestation of apathy. Moreover, structural differences in grey and white matter of the brain have been associated with higher apathy in the healthy population^6, 7, 9^. However, whether variations in apathy in the non-clinical population are also underpinned by functional abnormalities in regions subserving intentional behaviour has not been studied to date.

It has been suggested that apathy is not a unitary concept, but that it can be divided into different domains, including an emotional, cognitive, and auto-activation domain^2, 10, 11^. First, the emotional domain is thought to relate to the appreciation of, and rewarding feelings associated with the outcome of undertaking future actions. Second, the cognitive domain relates to executive functions needed to realize an action, such as cognitive planning, calculating needed effort, and controlling action. Finally, the auto-activation domain relates to the actual initiation of planned behaviour, e.g. to start the motor program. Levy & Dubois^2^ have proposed that apathy may be related to specific neural substrates underlying these subtypes of disrupted processing, suggesting that differential neural pathways could lead to the same behavioural manifestation.

So far, the neural basis of apathy has been investigated in the context of reward processing and executive control, mainly in clinical samples^12–19^ and less frequently in healthy individuals^7^. In these healthy individuals, an association has been found between higher levels of apathy and increased effort sensitivity, and between apathy and increased involvement of the supplementary motor area (SMA) and anterior cingulate cortex (ACC) and reduced connectivity between these brain regions^7^. To date, to our knowledge, the neural underpinnings of apathy related to self-initiation of actions independent of reward and effort computation have not been studied as yet, neither in patients nor in a healthy population.

Previous research on self-initiated behaviour in healthy individuals suggested that self-initiated behaviour is associated with the recruitment of fronto-parieto-striatal regions^1^. Separate components of self-initiated behaviour have been studied, including a selection component (i.e. deciding *what* action to perform), and a timing component (i.e. deciding on *when* to initiate a pre-specified or self-chosen action). Using functional Magnetic Resonance Imaging (fMRI), *what* and *when* components of action execution have been studied in healthy individuals employing a task that evoked either self-initiated, or externally triggered finger movements^20^. In this study, selecting which action to perform was associated with activation in medial frontal regions including the bilateral pre-supplementary motor cortex extending to the anterior midcingulate cortex, in addition to dorsolateral prefrontal cortices (DLPFC), dorsal premotor cortices, and inferior parietal lobules (IPL). Deciding on the timing of action execution was associated with largely overlapping regions, however with additional recruitment of the bilateral anterior insula, anterior putamen, globus pallidi, and left cerebellum^20^. Taken together, primary and supplementary motor regions, the DLPFC, ACC, IPL, and (parts of) the striatum have been consistently related to selection and timing components of action and therefore may have high relevance for disturbances in self-initiated behaviour underpinning apathy. Indeed, substantial evidence was found for consistent involvement of the ACC and IPL in a dysfunctional fronto-parieto-striatal network, in relation to apathy across disorders^21^. These results suggest that regions that were associated with apathy are largely in accordance with those involved in self-initiation of actions.

The aim of this paper was to investigate whether levels of apathy in a healthy population were associated with neural correlates of action initiation of self-selected behaviour. To this end, we employed an event-related functional MRI paradigm adapted from Hoffstaedter *et al*.^20^ that allowed us to investigate both the action selection and timing components of self-initiated behaviour. We hypothesized that higher levels of apathy would be related to altered activation of regions associated with intentional behaviour, namely within the fronto-parieto-striatal circuit. We expected apathy-related variations primarily during the condition where both type and timing of action could be freely determined. Furthermore, we hypothesized that higher levels of apathy would be associated with longer times needed to make a decision on what and when to act and with reduced variability in behaviour, i.e. more similar button presses. Finally, we tested whether levels of apathy could be predicted from multivariate patterns of brain activation during intentional behaviour using multivariate pattern analysis.

## Results

### Behavioural data

**Figure 1:**
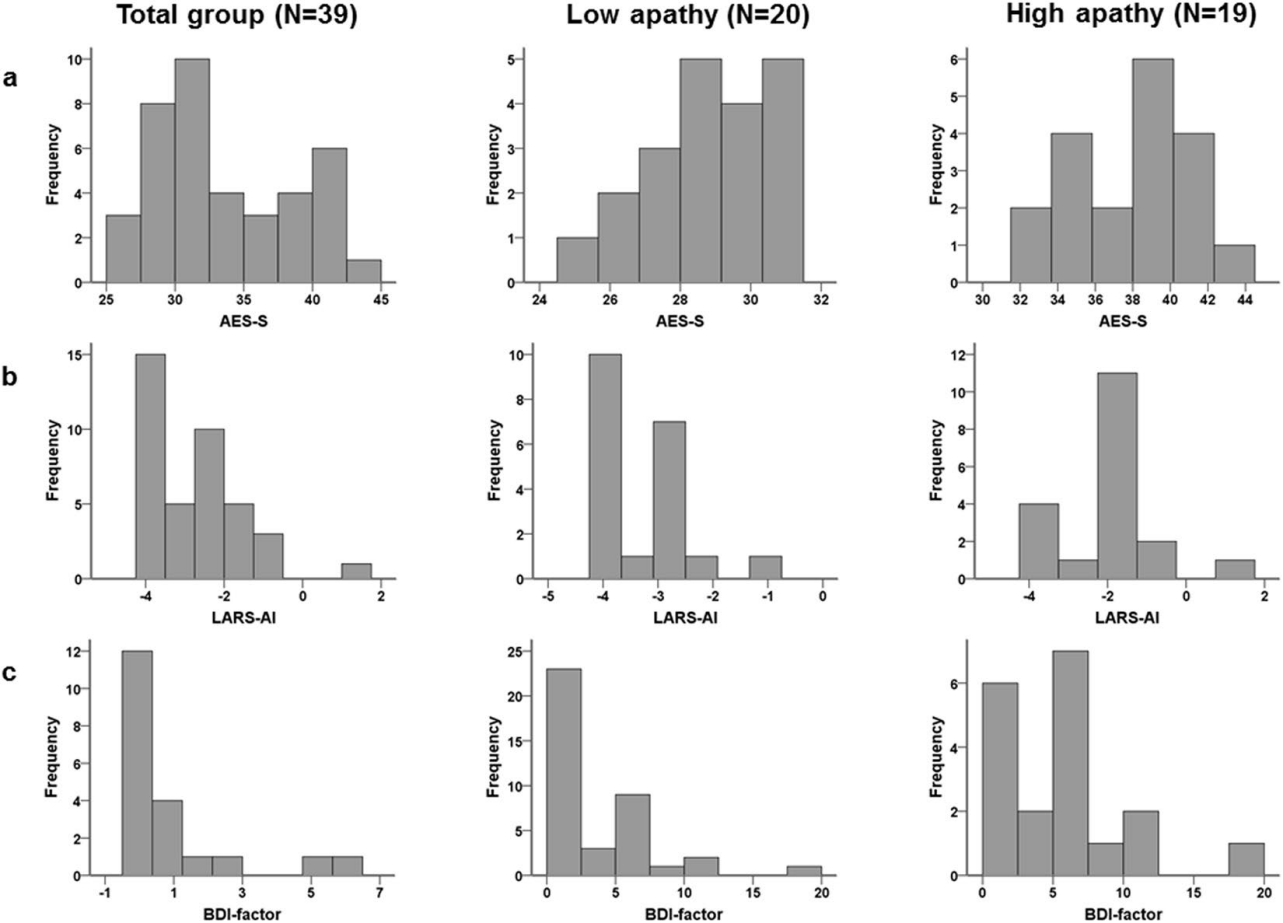
(**a**) Distribution of the AES-S of the total group (N = 39), the low apathy group (N = 20), and high apathy group (N = 19); (**b**) Distribution of the LARS-AI of the total group (N = 39), the low apathy group (N = 20), and high apathy group (N = 19); (**c**) Distribution of the BDI-factor of the total group (N = 39), of the low apathy group (N = 20), and high apathy group (N = 19).

Apathy, as measured with the self-rated version of the Apathy Evaluation Scale (AES-S), was significantly associated with action initiation as measured using the Lille Apathy Rating Scale (LARS_AI, Table 1). Furthermore, higher levels of apathy were significantly associated with higher depression scores (Beck Depression Inventory [BDI]), reduced pleasure (Temporal Experience of Pleasure Scale [TEPS], and Snaith-Hamilton Pleasure Scale [SHAPS]), higher (positive and negative) schizotypal symptoms (Schizotypal Personality Questionnaire [SPQ]), and higher symptoms of psychopathology (Symptom Checklist [SCL-90], Table 1, *p* < 0.05). Of note, the mean scores on the BDI, TEPS, SHAPS, SPQ, and SCL-90 were low and comparable to other normal populations, while the mean score on the AES-S was in the range of clinical populations^22^. Figure 1(a–c) displays the distribution of the AES, LARS-AI and BDI-factor for the total sample, as well as divided in two groups of low and high apathy scores.

**Table 1:** Demographical information and mean scores on the questionnaires for the total group and separate for participants scoring low and high on apathy.

Variables	Possible Range^1^	Total group	Total group	Total group	Low apathy	Low apathy	High apathy	High apathy
		Mean (SD) (N = 39)	Min/Max	τ with AES-S^2^	Mean (SD) (N = 20)	Min/Max	Mean (SD) (N = 19)	Min/Max
Age	—	22.69 (2.27)	18/27	—	22.75 (2.15)	19/27	22.63 (2.45)	18/26
Sex (M/F)	—	—	13/26	—	—	5/15	—	8/11
Education^3^	—	17.36 (1.89)	14/24	—	17.55 (2.16)	14/24	17.16 (1.57)	14/20
AES-S^×^	18/72	33.41 (5.33)	25/44	—	29.05 (1.79)	25/31	38 (3.64)	32/44
LARS-AI^×^	−1	−2.68 (1.26)	−2.6667	0.53**	−3.3 (0.86)	4	−2.03 (1.31)	−2.6667
BDI^×^	0/63	6.21 (6.35)	0/27	0.53**	2.55 (3.27)	0/12	10.05 (6.6)	0/27
BDI-factor^×^	0/33	3.28 (4.23)	0/19	0.51**	1 (1.75)	0/6	5.68 (4.76)	0/19
TEPS-ANT^×^	10/60	43.26 (6.92)	25/55	−0.43**	46.8 (5.24)	33/55	39.53 (6.59)	25/55
TEPS-CON^×^	8/48	38.69 (5.11)	26/47	−0.32*	40.7 (4.85)	31/47	36.58 (4.59)	26/47
SHAPS^×^	0/14	1.08 (2.6)	0/14	0.33**	0.9 (3.16)	0/14	1.26 (1.91)	0/7
SPQ-pos^×^	0/46	7.87 (5.96)	0/23	0.35**	5.35 (4.34)	0/14	10.53 (6.36)	23-Feb
SPQ-neg^×^	0/43	8.05 (5.86)	0/22	0.58**	4.4 (3.35)	0/11	11.89 (5.56)	22-Feb
PANAS-pos^×^	10/50	32.41 (7.6)	15/46	−0.38**	35.85 (5.31)	26/46	28.79 (8.07)	15/44
PANAS-neg^×^	10/50	14.21 (3.68)	10/25	0.18	12.7 (2.58)	10/18	15.79 (4.05)	10/25
SCL-90^×^	90/450	121.49 (24.76)	92/179	0.46**	108.25 (14.81)	95/157	135.42 (25.74)	92/179

*p < 0.05 is statistically significant. ** Significant after Bonferroni correction, *p* < 0.005.

^×^Significant different means for low and high apathy groups, t-test, *p* < 0.05.

AES-S = Apathy Evaluation Scale, Self-rated; BDI = Beck Depression Inventory; PANAS = Positive and Negative Schedule; LARS_AI = Lilly Apathy Rating Scale, Action Initiation subscale; SCL-90 = Symptom Checklist; SHAPS = Snaith-Hamilton Pleasure Scale; SPQ = Schizotypal Personality Questionnaire; TEPS = Temporal Experience of Pleasure Scale; TEPS-ANT = Anticipatory pleasure subscale of the TEPS; TEPS-CON = Consummatory subscale of the TEPS.

^1^A higher number indicates higher severity, except for PANAS-pos and TEPS.

^2^Correlations between apathy scores and other factors are calculated with a Kendall’s Tau test (τ).

^3^Years including primary school.

### Neuroimaging results

#### Overall task effects

**Figure 2:**
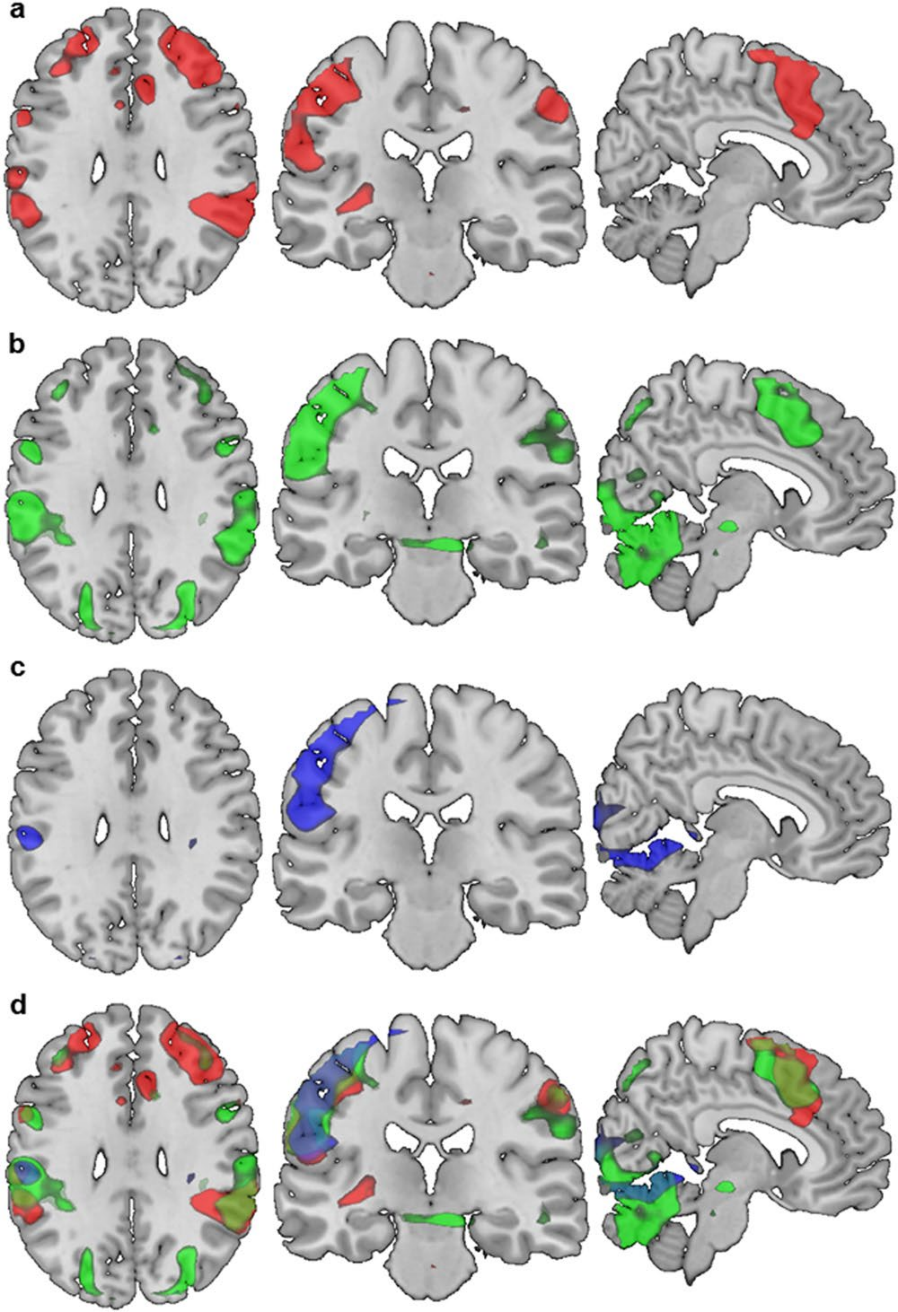
Whole-brain task activation of all participants (N = 39) during the (**a**) free condition (red), (**b**) timed choice condition (green), (**c**) no choice condition (blue), and (**d**) free, timed choice, and no choice conditions, all significant p < 0.05 FWE cluster-corrected (initial threshold p < 0.001, uncorrected). Coordinates (MNI): x = 6, y = −22.5, z = 30.

Activation elicited in the *free* condition (i.e. freedom in timing and selection of actions) compared to the implicitly modelled low level baseline (i.e. fixation cross) was primarily found in bilateral dorsolateral and ventrolateral prefrontal regions, inferior frontal gyri, insula, precentral and postcentral gyri, anterior and midcingulate cortex (ACC and MCC), supramarginal gyri, inferior parietal lobes (IPL), cerebellar crura, and anterior cerebellar regions (extending to the left lingual gyrus and right fusiform gyrus). Additional activation during the *free* condition was found in the occipital lobe, right precuneus, and right superior parietal lobe (Fig. 2a and Supplementary Table S1 for all peak activations). Higher activation in the *timed choice* condition (i.e. freedom in selection of actions) compared to low level baseline was primarily found in the right ventrolateral, dorsolateral and inferior orbital frontal regions, left inferior operculum (extending to the insula), left precentral gyrus, bilateral midbrain, bilateral middle and inferior occipital regions, and the anterior cerebellum. A single cluster of lower activation (compared to baseline) was found in the bilateral medial orbitofrontal gyrus (Fig. 2b and Supplementary Table S1 for all peak activations). Lastly, increased activation elicited by the *no choice* condition (i.e. no freedom in timing nor selection of actions) was primarily found in the left supramarginal gyrus extending to the IPL and precentral gyrus, the left operculum, and furthermore right occipital and anterior cerebellar regions (Fig. 2c and Supplementary Table S1 for all peak activations).

#### Association with apathy

No relations were found between apathy and activation during *free*, *timed choice*, and *no choice* conditions, nor when investigating more complex contrasts evaluating *what* action to perform and *when* a to initiate a pre-specified action, in regression and group analyses, for both voxel-based ROI-constricted and whole-brain analyses at *p* < 0.05 FWE corrected at cluster level (initial threshold of *p *< 0.001, uncorrected). Uncorrected peak activations of the Self-Initiative task ROI-analyses for the *free*, *timed choice*, and *no choice* conditions, for the total group and high versus low apathy groups are displayed in Supplementary Table S2. Adding BDI, SPQ, and SHAPS scores to the model as covariates to account for variations in depression severity, psychopathy and hedonic capacity, did not change these results. Excluding participants with mild to moderate depression (BDI > 13) also did not change the results. Lastly, no associations were found between apathy severity and behavioural responses of the self-initiative task (see Supplementary Material and Supplementary Table S3).

In exploratory multivariate regression analyses, correlations between observed and predicted apathy scores were non-significant for the *free* (r = 0.21, *p* = 0.09; R^2^ = 0.04, *p* = 0.46; MSE = 28.83, *p* = 0.10), *timed choice* (r = 0.05, *p* = 0.22; R^2^ = 0.00, *p* = 0.87; MSE = 34.78, *p* = 0.68), and *no choice* contrasts (r = −0.29, *p* = 0.73; R^2^ = 0.09, *p* = 0.33; MSE = 40.67, *p* = 0.90), indicating that degree of apathy could not be predicted based on the activation pattern in grey matter voxels. Furthermore, SVM analyses revealed that the automated classifier only performed at chance to differentiate the two apathy groups for all three conditions (classification accuracies are displayed in Supplementary Table S4).

## Discussion

To our knowledge, the current study is the first designed to investigate the neural underpinnings of apathy in a non-clinical sample by focussing on self-initiated behaviour. We included participants with apathy ranging from low to high scores, and manipulated the extent to which they could freely execute finger movement behaviour while measuring brain activation using functional MRI. The self-initiative task robustly activated fronto-parietal regions when participants decided what action to perform and when to perform it. However, no relationship between degree of apathy and brain activation during action initiation was observed, neither when mass-univariate analysis was used, nor when multivariate patterns of brain activation were considered. In summary, in this study no correlation was found between apathy in a student sample and neural substrates related to auto-initiation components of action execution.

In the current study, apathy was studied in relation to goal-directed behaviour by focusing on self-initiation of simple finger movements in a controlled experimental task. This way, the effects of emotional or hedonic components that are commonly part of goal-directed action could be minimized, isolating the possible effects of disrupted action initiation of apathy. We hypothesized that apathy was at least in part underpinned by deficits in an action-initiation circuit and specifically hypothesized that higher apathy scores would be related to lower variability in choice of action and less recruitment of fronto-parietal areas important for action initiation. However, these hypotheses were refuted by the data. This either means that the neural correlates of self-initiated action are indeed not involved in apathy in a non-clinical sample, or that our findings can be explained by methodological drawbacks or artefacts. Therefore, several possible alternative explanations for our findings will be discussed successively.

First of all, the lack of associations between apathy and behavioural and neural measures of self-initiation may raise the question whether the measurement of apathy was valid and reliable in this study. Apathy was measured with the Apathy Evaluation Scale, self-rated version (AES-S), which is a standardized, reliable, questionnaire that is suitable for a non-clinical population, though most frequently used in clinical populations, e.g. patients with schizophrenia^23^. The self-rated version of the AES has been demonstrated to have lower correlations with behavioural measures compared to the AES rated by a clinician (AES-C) or informant (AES-I) in a validity study. Therefore, it could be possible that the AES-C and AES-I are more sensitive in measuring apathy, but this needs further study. Moreover, the lower correlations have been attributed to a relatively narrow range of AES-S scores in that particular sample^23^, while in the current study a wide variety in scores was observed, bolstering confidence in the sensitivity of our measurements. Furthermore, in our sample, AES-S scores were associated with reduced action initiation of daily life activity as measured with a clinical apathy evaluation instrument (i.e. the LARS_AI), but also with reduced pleasure (as measured with the SHAPS and TEPS), and higher depression (BDI), supporting convergent validity. Even though correlations between these measures of apathy, pleasure, and depression were significant and indicated an overlap in symptoms, the correlation coefficients (ranging between 0.32 and 0.53) also demonstrated that the AES-S measures a unique aspect that is not incorporated in other questionnaires, supporting discriminant validity. In other words, the symptoms measured by the AES-S might overlap with depressive or anhedonic symptoms, e.g. staying in bed or indoors and reduced feelings of anticipatory pleasure, but the AES-S also measures symptoms that are more specific to apathy, i.e. motivational and self-initiation aspects of behaviour. In our MR-analyses we entered the AES-S and additional covariates in order to specifically evaluate the relationship between neural correlates and the self-initiation component of apathy, while taking into account the possible effects of mood and pleasure (or anhedonia). However, accounting for variations on other clinical measures and even excluding participants with signs of mild to moderate depression did not change the results.

Secondly, it is important to consider what exactly is being measured by the self-initiative task that we employed. Levy & Dubois^2^ described the auto-initiation deficit as “difficulties in activating thoughts or initiating the motor program necessary to complete the behaviour”. A person suffering from an auto-activation deficit particularly has problems in self-initiation of actions, while externally driven responses and actions are spared. In the task we used, both aspects were acknowledged; we attempted to provide circumstances in which participants were indeed free in their choice and timing of actions, but also conditions in which a person was provided with a structured assignment and whereby behaviour was more externally driven. Using a paradigm that provides assignments to act voluntary might however be regarded as paradoxical. The range of possible behavioural responses in our paradigm was limited and might even in the most free condition be considered as externally driven or comparable to a decision making task because we provided the subjects with a limited range of possible behaviours. Nevertheless, according to Haggard (2008), these paradigms “capture a key computational feature of voluntary action, namely the participant must themselves generate the information that is needed to perform an action”. Our study is in line with this idea. However, we need to keep in mind that we studied self-initiation in a limited and perhaps artificial way within a controlled setting.

Moreover, brain activation related to the task corresponded with different stages of self-initiation, which provides further support for the validity of the paradigm that was used. In most restricted assignments without freedom in selection and timing of behaviour (the *no choice* condition), particularly regions related to motor behaviour (e.g. planning, intention, motor speed, and control) were involved, including the precentral gyrus, inferior parietal, and cerebellar regions, which is in accordance with the existing literature^20, 24–28^. The paradigm also reliably activated expected regions as a function of increasing task load. With increasing task freedom, we found activation in regions related to executive processes, attentional control, and decision making, including parietal regions, insula, anterior and midcingulate regions, and dorsolateral and inferior frontal regions^29–33^. In assignments offering more freedom in timing and selection of actions similar regions were involved, albeit to a larger extent, and more bilaterally distributed.

Another aspect of the self-initiative task that warrants discussion regards its specificity. A clear advantage of this task is that it does not involve any reward components, effort computation, and only minimally involves memory and planning components (functions related to the cognitive and affective domains of apathy), which makes it a task that is specifically aimed at auto-activation of movement. Of note, studies that reported behavioural aspects to be involved in apathy in the healthy population have thus far always employed tasks that included rewarding components and effort computation, which might impede drawing conclusions on selective behavioural aspects of apathy.

Our results thus rather suggest that apathy in non-clinical populations is not strongly underpinned by core abnormalities in initiating motor behaviour. It is perhaps more likely that in the healthy population apathy, as a multidimensional construct, is stronger associated with complex processes including effort computation, planning and reward learning, as was previously demonstrated in other studies^7, 34, 35^. We observed that participants with higher levels of apathy presented reduced pleasure, primarily in the anticipatory phase compared to in-the-moment consummatory pleasure, which is in accordance with anhedonia in patients with schizophrenia suffering from apathy^12^ and anhedonia in healthy university students^36^. Effort computation was however not examined in our population, nor were the cognitive aspects of apathy, which therefore limits further conclusions on apathy-related deficits in our studied sample.

For future studies, it would be interesting to evaluate the neural correlates of apathy in other samples, including clinical populations or individuals with lower education and higher age, as one might expect that levels of apathy may be higher in these populations. Furthermore, where in the current study there was insufficient information on stability of apathy severity over time within our included participants, both individuals with stable, long-term apathy (‘trait’ apathy), as well as those with temporary or adaptive apathy (‘state’ apathy) may have been included. Of note, more chronic or severe forms of apathy may be less likely in our university student sample, in contrast to apathy induced by stressful changes in the social or physical environment of a person. The results from the present study might suggest that apathy as a non-clinical behavioural manifestation maybe qualitatively different from apathy as presented in clinical populations (i.e. patients with schizophrenia, Parkinson’s Disease), but further work is required to establish the viability of this suggestion.

In conclusion, in this study degree of apathy as measured with a clinical apathy evaluation scale was not associated with activation of brain regions in the fronto-parieto-striatal circuit during an fMRI task that evoked self-initiated behaviour in a highly-educated sample of young individuals. These results suggest that alterations in starting motor programs, a critical component of the auto-activation subdomain of apathy, do not explain the occurrence of apathy in the normal population. According to Levy & Dubois^2^, the auto-activation component of apathy is the most severe form of apathy, which frequently occurs in patients with focal basal ganglia lesions. Therefore, it might be suggested that this component is not, or not strongly, involved in a population without psychiatric and neurological complaints. This may also explain why they can still function relatively well, i.e. they did not seek professional help for their apathy (and did not receive a neurological or psychiatric diagnosis) as it may have been less disruptive to their daily life because of intact action-initiation.

## Methods

### Participants

For this study, university and vocational university students were recruited via advertisements on university websites, via email, posters, and by word of mouth. In total, 469 students responded to our advertisements, and subsequently a pool of 300 students completed the Apathy Scale (AS)^37^ and a short questionnaire regarding the inclusion criteria, and MR safety. From this initial sample, participants with the highest (N = 20) and lowest scores (N = 20) were selected to assure sufficient variability in apathy scores. Participants with high and low scores were matched on age and sex. Participants were native Dutch speakers, right-handed, and MR-compatible. They did not report any neurological or psychiatric disorders or visual or hearing problems that could not be corrected, and did not take medication that could influence task performance. Participants were invited to complete an fMRI protocol including an anatomy scan and three tasks, among which a self-initiation task. Time between initial sign-up and invitation to complete the fMRI protocol ranged between two and 13 months. All participants gave informed consent after having received written information about the aims and procedures of the study. The study protocol was approved by the local medical ethical committee of the University Medical Center Groningen. The procedures were carried out according to the latest version of the declaration of Helsinki^38^.

### Behavioural measurements

At time of MR data acquisition, several measures were used to quantify behavioural characteristics of the selected participants (N = 40). The self-rated Apathy Evaluation Scale (AES-S)^23^ and the semi-structured interview for the Lille Apathy Rating Scale (LARS)^39^ were used to measure apathy. Apathy measured with the AES at time of MR-scanning correlated significantly with apathy measured with the AS during the screening phase (τ = 0.63, *p* < 0.001 [one-tailed]). The AES-S was considered our primary outcome measure because we believed it was better suitable for the population we investigated. However, the Action Initiation (AI) subscale of the LARS was used to measure everyday productivity and self-initiation to specifically characterize self-initiation aspects of goal directed behaviour. To further investigate characteristics of our sample, the Snaith-Hamilton pleasure scale (SHAPS)^40^ and Temporal Experience of Pleasure Scale (TEPS)^36^ were assessed to measure the degree to which an individual is capable to experience pleasure. To evaluate the general psychological status of the included participants, the Beck Depression Inventory (BDI)^41^, the Symptom Checklist 90 (SCL-90)^42^, the Schizotypal Personality Questionnaire (SPQ)^43^, and the Positive and Negative Affect Schedule (PANAS)^44^ were added to the protocol. Because depressive and apathetic symptoms partly overlap, we separately mention the score on the core “depressed mood” items of the BDI, previously identified in a meta-analysis of factor structures^45^.

### Task design

**Figure 3:**
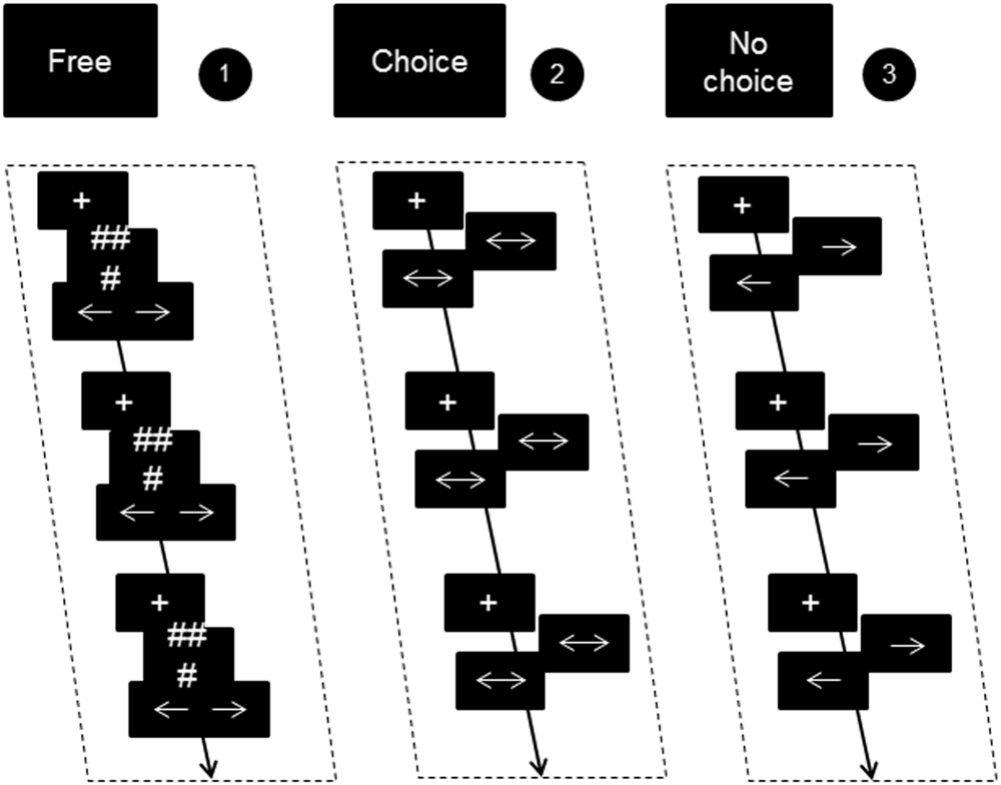
Outline of the Self-Initiative task with three conditions that were pseudorandomized in blocks (adapted from Hoffstaedter *et al*.^20^).

The task that was used is based upon the self-initiative task developed by Hoffstaedter *et al*.^20^, including adjustments on response options and duration of the task. This task was designed to evoke self-initiated behaviour, by allowing participants to select what to do and when to act. During the task, participants were asked to take initiative by pressing one of two buttons with their right index or right middle finger at a visual cue or at a self-chosen point in time. The task consisted of three conditions: *free*, *timed choice*, and *no choice* (see Fig. 3). In the *free* condition, participants could choose which button to press and when to press it. The *timed choice* condition only offered freedom in choosing which button to press, on a fixed point in time. In the *no choice* condition participants were requested to respond with a fixed button press (i.e. left or right) as quick as possible after a cue. During instructions participants were asked not to provide rhythmic or routine responses in the *free* and *timed choice* conditions. Comparison of the conditions allowed for examination of brain activation related to the what and when components of self-initiated behaviour.

### Free choice

During *free* trials, participants were presented with a visual cue (hashes) during which the participant had to choose to press the left or right button at any time, though when no button press was recorded within 20 seconds, the next trial was presented. After a button press, the participant received feedback on which button was pressed, by means of an arrow which was presented for 3.5 seconds. In case of no button press, a message that no response was recorded was shown. Participants were instructed not to press a button during feedback. Afterwards, a fixation cross appeared (500 ms) which was followed by the next trial. Response times within this condition were used to determine the interstimulus intervals (ISIs), i.e. the duration of the fixation crosses, for the subsequent *timed choice* and *no choice* conditions, in order to keep the number of trials equal for all three conditions. Response times were calculated using the onset of the visual cue and subsequent button press as offset.

### Timed choice

During the *timed choice* condition, a double arrow (pointing to the left and right side of the screen) was presented as a cue during which the left or right button (as chosen by the participant) had to be pressed as quickly as possible. The double arrow was visible for 3.5 s. In between trials a fixation cross was presented. The durations of these fixation crosses (ISI’s) varied, and were based upon the response times during the *free* condition (presented in random order).

### No choice

During the *no choice* condition an arrow was presented, which pointed either to the left or the right side of the screen. Participants had to respond as quickly as possible by pressing the corresponding button. The arrow remained visible for 3.5 s. In between the arrows, fixation crosses were presented of which the length varied and was determined by the response times during the *free* condition.

The task consisted of five blocks in total, and each block comprised the three conditions (*free*, *timed choice* and *no choice*), presented in sub blocks of 60 seconds each and separated by 15-second periods of fixation. The five blocks lasted 210 seconds each, with 8–9 s fixation periods in between the blocks, in addition to a 20 s-fixation period at the beginning and a 8–19 s fixation period at the end of the task, which adds up to a total task duration of 18–22 minutes. Variations in fixation periods can be explained by programming in TRs, not in seconds. The order of the sub blocks alternated between ‘*free*-*timed choice*-*no choice*’ and ‘*free*-*no choice*-*timed choice*’. Because ISI’s in the *timed choice* and *no choice* conditions were determined by the response times in the *free* condition, the latter condition was always presented first. In accordance with Hoffstaedter *et al*.^20^, we did not introduce conditions that separately manipulated the when (timing) component.

### Image acquisition

Imaging data was acquired on a 3.0 Tesla magnetic resonance imaging system (Philips Intera, Best, The Netherlands) equipped with a 32-channel SENSE head coil. For anatomical reference, a T1-weighted image was obtained (TR/TE = 9/3.5 ms) using fast-field echo and turbo-field echo: 170 axial slices; FOV (rl, ap, fh) = 232 × 170 × 256 mm; flip angle = 8°, voxel size = 1 × 1 × 1 mm, slice thickness = 1 mm. An Echo Planner Imaging sequence was used for functional scanning (TR/TE = 2000/22 ms) using 47 descending axial slices; FOV (rl, ap, fh) = 192 × 192 × 141 mm, flip angle of 90°, voxel size = 3 × 3 × 3 mm, slice thickness = 3 mm; slice gap = 0 mm.

### Demographic and behavioural data analyses

Demographic and behavioural data was analysed using IBM SPSS Statistics (Version 23) and MATLAB 2013a (The Mathworks, Natick MA, USA). The independent variable apathy, as measured with the AES-S, was treated as a continuous variable because the distribution was not deviant from a unimodal distribution (Hartigan’s dip test for unimodality^46^ D = 0.06, *p* = 0.2). However, because the assumption of normality was not met for AES-S scores (Shapiro-Wilk W test = 0.924, *p* = 0.011), possible associations between demographic variables and apathy were tested with the non-parametric Kendall’s Tau correlation measure. Results were considered significant for *p* < 0.05.

### Functional magnetic resonance imaging

fMRI data were analysed in the context of the General Linear Model (GLM) using Statistical Parametric Mapping (SPM12; version 6470; http://www.fil.ion.ucl.ac.uk/spm/software/spm12/), in MATLAB 2013a (The Mathworks, Natick MA, USA). Before processing the functional images, we performed visual inspection to check for possible artefacts. Further, image origins (of T1 and EPI- images) were manually set to the centre point of the anterior commissure to ensure proper alignment with the templates. Preprocessing steps included correction for slice timing, realignment, co-registration of the T1-image to the mean functional image, normalization to Montreal Neurological Institute (MNI) space by warping the co-registered T1 image to SPMs standard Tissue Probability Map template in standard space and that transformations were then applied/written to the functional images, and smoothing with an 8 mm isotropic Full Width at Half Maximum (FWHM) Gaussian Kernel. Data for one participant was excluded due to excessive movement during the entire experiment. Movement was deemed excessive if participants moved more than 3 mm in x, y, or z direction or if rotations were more than 1 degree in any direction.

In accordance with Hoffstaedter *et al*.^20^, the self-initiative task was modelled in an event-related manner for the conditions *free*, *timed choice*, and *no choice*. We employed presentation (hashes/arrows) to define trial onsets, and response times were used to define the duration of each event for the three conditions (*free*, *timed choice*, and *no choice*). Non-response trials and sudden peaks in head motion ( > 3 mm or 1 degree in any direction) were modelled as separate regressors of no interest. Furthermore, motion parameters and their first derivatives were added to the model (i.e. 12 motion regressors in total).

### Statistical modelling of fMRI-data

At first level, contrasts were defined for each condition (*free*, *timed choice*, and *no choice*). At second level, *free*, *timed choice*, and *no choice* contrasts were entered into a one-sample T model to evaluate task related activity vs. activity at low level implicit baseline (i.e. during fixation crosses). Furthermore, multiple regression analyses were conducted to evaluate the relationship between AES-S score and brain activation on *free*, *timed choice*, and *no choice* contrasts. More complex contrasts as defined by Hoffstaedter *et al*.^20^ (*what* and *when*), were unsuitable to define at first level because we observed an inequality of residual means squares over the three conditions, leading to an underestimation of task effects at the second level (see Supplementary Fig. S1). Therefore, these complex contrasts were not included in the regression analyses.

Regression analyses were considered the most appropriate statistical method because of the unimodal distribution of AES-S scores in the included sample and therefore mass-univariate regression analyses were considered our main analyses. However, because participants were initially selected on low and high apathy scores, additional exploratory group analyses were performed. For this reason, *free*, *timed choice*, and *no choice* contrasts were entered into two-sample T models to evaluate possible differences between low and high apathy severity groups (based on a median split). Moreover, a flexible factorial model was applied to evaluate group differences on more complex contrasts, which allowed to properly handle the inequality in residual means squares of the conditions. This model included group (high and low apathy) as between-subject factor and condition (*free*, *timed choice*, and *no choice*) as within-subject factor, which allowed for assuming unequal variances in the condition factor. In this model, group differences for more complex contrasts, to separate the ‘when’ and ‘what’ component of self-initiated behaviour (see Hoffstaedter *et al*.^20^) were explored. These contrasts allowed to directly compare *free* with *timed choice* and *no choice* conditions ([Free > Timed Choice] ∩ Free > No Choice]), to separate the ‘when’ component of self-initiated behaviour and the *timed* and *free* with the *no choice* condition ([Timed Choice > No Choice] ∩ Free > No Choice]) to separate the ‘what’ component of self-initiated behaviour.

In a subsequent step of the main regression analyses, hedonic capacity (SHAPS), depression (BDI core depression score), and positive schizotypal symptoms (SPQ-pos) were included as covariates in the second level models. Hedonic capacity was included to regress out variance in the AES-S scores that related to the emotional aspects of apathy (lowered propensity to anticipate to and experience pleasurable feelings). This way, we studied the relationship between self-initiation related brain activation and AES-S score including the cognitive and behavioural components of apathy. Furthermore, core depressive symptoms and positive schizotypal symptoms were included as covariates, because these symptoms may result in behaviour that resembles apathetic behaviour (e.g. staying indoors, reducing social contact). Furthermore, in order to evaluate possible neural correlates of apathy independent of depression, a last additional analysis was performed only including participants that scored “minimal” on depression (range 0–13; excluding N = 5 for BDI > 13).

All voxel-wise analyses were performed both at whole-brain level and using a ROI-restricted approach. Our ROI mask included regions that were previously found to be related to apathy^21^, as well as the self-initiative task that we used and included large portions of the inferior and middle frontal gyri, (pre) supplementary motor cortex, premotor cortex, inferior parietal cortex, supramarginal gyrus, cingulate cortex, thalamus, amygdala, striatum, globus pallidus, hippocampus, and some regions within temporal lobe (see Supplementary Fig. S2).

The threshold was set at *p* < 0.05, family wise error (FWE) corrected at cluster level with an initial threshold of *p* < .001, uncorrected. For our regions of interest, the correction area was restricted to the spatial extent of the composite mask. For regions outside our ROI mask, a correction for the whole brain was applied.

### Multivariate analyses

Additional exploratory analyses were performed in the context of multivariate regression and group classification using the “Pattern Recognition for Neuroimaging Toolbox^47^. First, three separate multivariate regression analyses were performed for the *free*, *timed choice*, and *no choice* contrasts (vs. implicit baseline) to investigate the potential of whole-brain functional images for predicting the severity of apathy using Relevance Vector Regression. Multivariate weight maps were constructed to visualize the spatial pattern driving the regression. Second, linear SVM learning was used to classify participants to the low and high apathy group. Prior to regression and SVM analyses, the contrast maps where masked using a standard grey matter mask.

To assess generalizability, a leave-one-out cross-validation (LOOCV) procedure was carried out. During this procedure, the analysis was repeated as many times as there were participants, excluding all data from a single subject at each iteration. Data for the remaining participants was subsequently used to train the model; the data for the excluded participant was used for testing the algorithms.

Statistical significance was assessed using permutation testing. AES-S scores and group classifications were randomly permuted 1000 times and the models were tested using these labels. The number of times the permuted accuracy was greater than the true accuracy was counted and divided by the number of permutations in order to produce a p-value.

### Ethical Approval

All procedures performed in studies involving human participants were in accordance with the ethical standards of the institutional and/or national research committee and with the 1964 Helsinki declaration and its later amendments or comparable ethical standards.

## Electronic supplementary material

Supplementary information

## References

[1] Haggard P (2008). Human volition: towards a neuroscience of will. Nat. Rev. Neurosci..

[2] Levy R, Dubois B (2006). Apathy and the functional anatomy of the prefrontal cortex-basal ganglia circuits. Cereb. Cortex.

[3] van Reekum R, Stuss DT, Ostrander L (2005). Apathy: why care?. J. Neuropsychiatry Clin. Neurosci..

[4] Spalletta G, Fagioli S, Caltagirone C, Piras F (2013). Brain microstructure of subclinical apathy phenomenology in healthy individuals. Hum. Brain Mapp..

[5] Simon JJ (2015). Reward System Dysfunction as a Neural Substrate of Symptom Expression Across the General Population and Patients With Schizophrenia. Schizophr. Bull..

[6] Pardini M (2016). Prevalence and cognitive underpinnings of isolated apathy in young healthy subjects. J. Affect. Disord..

[7] Bonnelle V, Manohar S, Behrens T, Husain M (2016). Individual Differences in Premotor Brain Systems Underlie Behavioral Apathy. Cereb. Cortex.

[8] Fervaha G, Zakzanis KK, Foussias G, Agid O, Remington G (2015). Distress related to subclinical negative symptoms in a non-clinical sample: Role of dysfunctional attitudes. Psychiatry Res..

[9] Spalletta G, Fagioli S, Caltagirone C, Piras F (2013). Brain microstructure of subclinical apathy phenomenology in healthy individuals. Hum. Brain Mapp..

[10] Stuss, D. T., van Reekum, R. & Murphy, K. J. Differentiation of states and causes of apathy in *The Neuropsychology of Emotion* (ed. Borod, J) 340–363 (New York, 2000).

[11] Starkstein SE, Petracca G, Chemerinski E, Kremer J (2001). Syndromic validity of apathy in Alzheimer’s disease. Am. J. Psychiatry.

[12] Waltz JA (2009). Patients with schizophrenia have a reduced neural response to both unpredictable and predictable primary reinforcers. Neuropsychopharmacology.

[13] Waltz JA (2010). Abnormal responses to monetary outcomes in cortex, but not in the basal ganglia, in schizophrenia. Neuropsychopharmacology.

[14] Waltz JA (2013). The roles of reward, default, and executive control networks in set-shifting impairments in schizophrenia. PLoS One.

[15] Park IH (2015). Altered cingulo-striatal function underlies reward drive deficits in schizophrenia. Schizophr. Res..

[16] Mucci A (2015). Is avolition in schizophrenia associated with a deficit of dorsal caudate activity? A functional magnetic resonance imaging study during reward anticipation and feedback. Psychol. Med..

[17] Simon JJ (2010). Neural correlates of reward processing in schizophrenia–relationship to apathy and depression. Schizophr. Res..

[18] Wolf DH (2014). Amotivation in Schizophrenia: Integrated Assessment With Behavioral, Clinical, and Imaging Measures. Schizophr. Bull..

[19] Liemburg EJ (2015). Neural correlates of planning performance in patients with schizophrenia–relationship with apathy. Schizophr. Res..

[20] Hoffstaedter F, Grefkes C, Zilles K, Eickhoff SB (2013). The “what” and “when” of self-initiated movements. Cereb. Cortex.

[21] Kos, C., Tol, M. J. v., Marsman, J. C., Knegtering, H. & Aleman, A. Neural correlates of apathy in patients with neurodegenerative disorders, acquired brain injury, and psychiatric disorders. Neurosci. Biobehav. Rev. **69**, 381-401 (2016). 10.1016/j.neubiorev.2016.08.012 27527825

[22] Marin RS (1991). Apathy: a neuropsychiatric syndrome. J. Neuropsychiatry Clin. Neurosci..

[23] Marin RS, Biedrzycki RC, Firinciogullari S (1991). Reliability and validity of the Apathy Evaluation Scale. Psychiatry Res..

[24] Desmurget M, Sirigu A (2012). Conscious motor intention emerges in the inferior parietal lobule. Curr. Opin. Neurobiol..

[25] Johnson-Frey SH, Newman-Norlund R, Grafton ST (2005). A distributed left hemisphere network active during planning of everyday tool use skills. Cereb. Cortex.

[26] Kroliczak G, Michalowski B, Kubiak A, Pawlak M (2015). Disentangling the neural bases of action intentions: evidence from fMRI studies. J. Vis..

[27] Turner RS, Desmurget M, Grethe J, Crutcher MD, Grafton ST (2003). Motor subcircuits mediating the control of movement extent and speed. J. Neurophysiol..

[28] Wenzel U, Taubert M, Ragert P, Krug J, Villringer A (2014). Functional and structural correlates of motor speed in the cerebellar anterior lobe. PLoS One.

[29] Bechara A, Damasio H, Damasio AR (2000). Emotion, decision making and the orbitofrontal cortex. Cereb. Cortex.

[30] Kringelbach ML (2005). The human orbitofrontal cortex: linking reward to hedonic experience. Nat. Rev. Neurosci..

[31] Rolls ET, Grabenhorst F (2008). The orbitofrontal cortex and beyond: from affect to decision-making. Prog. Neurobiol..

[32] Tanji J, Hoshi E (2008). Role of the lateral prefrontal cortex in executive behavioral control. Physiol. Rev..

[33] Kuhn S, Brass M (2009). When doing nothing is an option: the neural correlates of deciding whether to act or not. Neuroimage.

[34] Bonnelle V (2015). Characterization of reward and effort mechanisms in apathy. J. Physiol. Paris.

[35] Engel M, Fritzsche A, Lincoln TM (2015). Subclinical negative symptoms and the anticipation, experience and recall of emotions related to social interactions: An experimental study. Psychiatry Res..

[36] Gard DE, Gard MG, Kring AM, John OP (2006). Anticipatory and consummatory components of the experience of pleasure: A scale development study. Journal of Research in Personality.

[37] Starkstein SE (1992). Reliability, validity, and clinical correlates of apathy in Parkinson’s disease. J. Neuropsychiatry Clin. Neurosci..

[38] World Medical Association Inc (2009). Declaration of Helsinki. Ethical principles for medical research involving human subjects. J. Indian Med. Assoc..

[39] Sockeel P (2006). The Lille apathy rating scale (LARS), a new instrument for detecting and quantifying apathy: validation in Parkinson’s disease. J. Neurol. Neurosurg. Psychiatry..

[40] Snaith RP (1995). A scale for the assessment of hedonic tone the Snaith-Hamilton Pleasure Scale. Br. J. Psychiatry.

[41] Beck AT, Ward CH, Mendelson M, Mock J, Erbaugh J (1961). An inventory for measuring depression. Arch. Gen. Psychiatry.

[42] Derogatis LR, Lipman RS, Covi L (1973). SCL-90: an outpatient psychiatric rating scale–preliminary report. Psychopharmacol. Bull..

[43] Raine A (1991). The SPQ: a scale for the assessment of schizotypal personality based on DSM-III-R criteria. Schizophr. Bull..

[44] Watson D, Clark LA, Tellegen A (1988). Development and validation of brief measures of positive and negative affect: the PANAS scales. J. Pers. Soc. Psychol..

[45] Shafer AB (2006). Meta-analysis of the factor structures of four depression questionnaires: Beck, CES-D, Hamilton, and Zung. J. Clin. Psychol..

[46] Hartigan JA, Hartigan PM (1985). The Dip Test of Unimodality. Ann. Stat..

[47] Schrouff J (2013). PRoNTo: pattern recognition for neuroimaging toolbox. Neuroinformatics.

