# Supplementary material for: Neural basis of self-initiative in relation to apathy in a student sample
Source: Sci Rep. 2017 Jun 12;7:3264. doi: 10.1038/s41598-017-03564-5 (PMC5468419; doi:10.1038/s41598-017-03564-5)
Supplement: Supplementary file 1 — Supplementary information [file 41598_2017_3564_MOESM1_ESM.pdf]

Title: **Neural basis of self-initiative in relation to apathy in a student sample**

Authors: Claire Kos; Nicky G. Klaasen; Jan-Bernard C. Marsman; Esther M. Opmeer; Henderikus Knegtering; André Aleman; Marie-José van Tol

Supplementary Material

Behavioural task analyses

A repeated measures analysis of variance (ANOVA) was performed to evaluate whether response times over the three conditions were significantly different from each other. Furthermore, possible associations between apathy and behavioural responses during the fMRI task were tested by means of an analysis of covariance (ANCOVA) for the total group, and using two-samples T-tests for differences between low and high apathy groups. In the total group analysis, apathy was included as a covariate of interest and condition was included as a within-subject factor. Separate models were constructed for response times (including all three conditions *free*, *timed choice*, and *no choice*), proportion of left responses, and response variability (both for two conditions *free* and *timed choice*). The proportion of left button presses was calculated in percentages per condition. The variability in button presses was calculated in a binary fashion as follows: in case a response was different from the previous response (i.e. using a different finger) it was counted as 1, in case it was the same it was counted as 0. Response counts were summed and divided by the total number of responses per participant to obtain a measure of variability. The *no choice* condition was not included in the ANCOVA analyses, because response type was predetermined in this condition. The relationship between response accuracy (i.e. the number of correct button presses) in the *no choice* condition and apathy was evaluated with a Kendall's Tau test.

Behavioural task results

Repeated measures ANCOVA with apathy as a continuous covariate of interest did not reveal significant main effects of apathy on response times ($F(1, 37) = .18$, $p = .67$), proportion of left responses ($F(1, 37) = .01$, $p = .94$), or on response variability ($F(1,37) = 1.17$, $p = .29$). Furthermore, there were no significant interactions between apathy and condition (*free*, *timed choice*, *no choice*) on response times ($F(1, 37.04) = .08$, $p = .79$), proportion of left responses ($F(1, 37) = .41$, $p = .53$) and variability of responses in the *free* and *timed choice* conditions (Table 2, $F(1, 37) = .45$, $p = .51$). The correlation analysis showed no relationship between apathy and the accuracy of responses in the *no choice* condition (Supplementary Table S2, Kendall's $\tau = -.11$, $p = .35$). Lastly, all two-samples T-tests for group differences on the behavioural task results were non-significant.

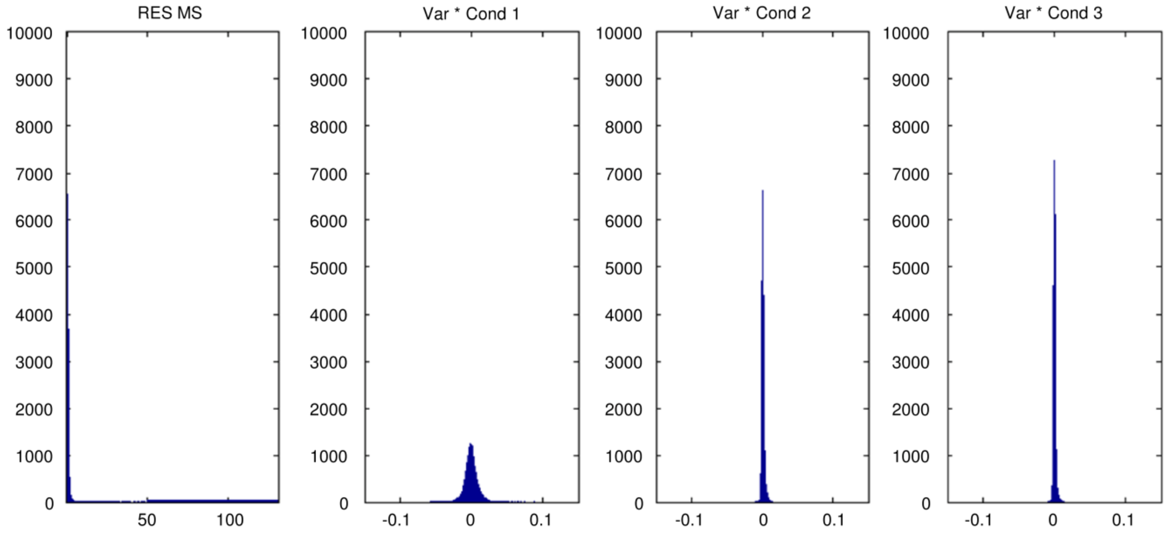

Supplementary Figure 1: Visualization of the residual means squares (ResMS) of one of the participants, representative for the pattern observed in all participants (cond 1= *free*; cond 2 = *timed*; cond 3 = *no choice*)

For all participants the distribution of the residual means squares (ResMS) of the *timed choice* and *no choice* conditions were comparable in height and shape, while the distribution of the ResMS of the *free* condition was lower and wider (for an example, see Figure S1).

We can only speculate on the reason of this difference in ResMS over the three conditions. It may be due to a more widespread activation pattern in the *free* condition, compared to the other conditions. It is also possible that estimation of the error is less accurate in the *free* condition. Because of the difference in distribution of the ResMS between the conditions, more complex contrasts including the *what* (timed & free > no choice) and *when* (free > timed & no choice) contrasts, as previously specified by Hoffstaedter et al. (2013), could not reliably be defined at the first level and therefore not entered in a Second-Level analysis.

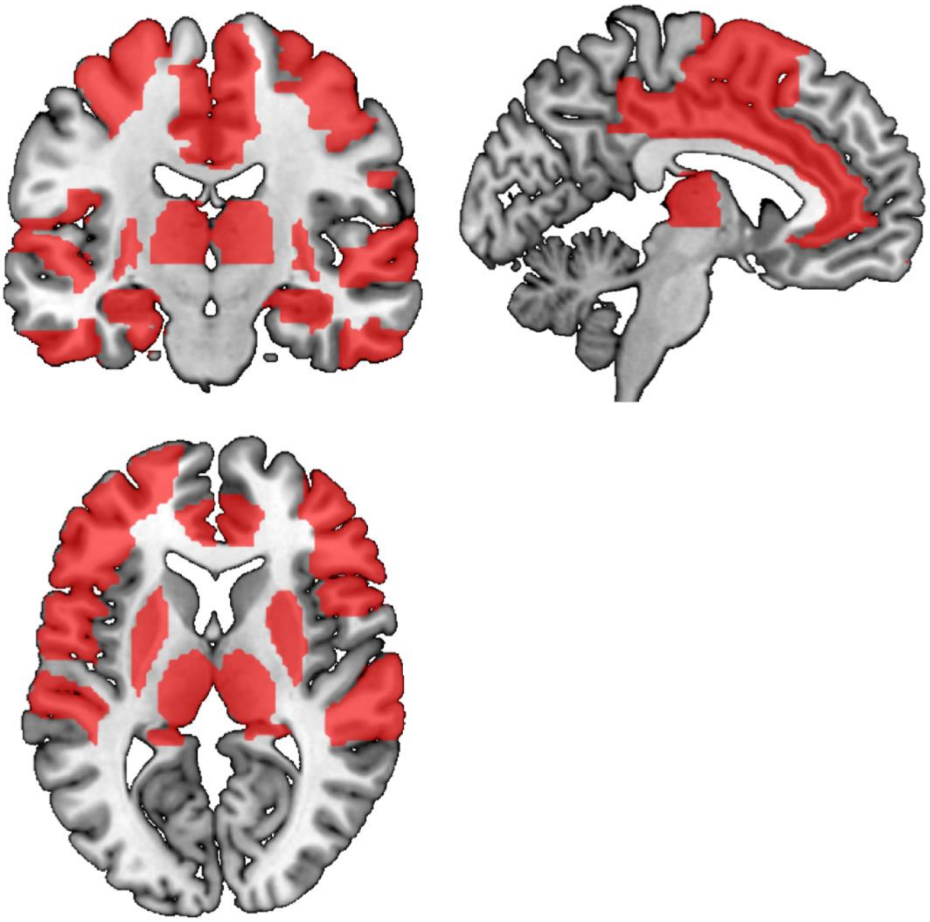

Supplementary Figure 2: The mask that was used in the Region of Interest (ROI) analyses. Coordinates: x = -5.5, y = -14.5, z = 8. The mask was composed using regions previously associated with apathy as well as with the self-initiative task. To define these overlapping regions, a composite mask was built using two separate binarized masks; one 'apathy mask' based on Kos et al. (2016) and one 'self-initiative mask' for current task-activation summed over *free*, *timed choice*, and *no choice* contrasts at p <.005 uncorrected and k > 10). Both binarized masks were multiplied using the Imcalc function in SPM12, to only end up with regions present in both masks. Lastly, all regions of the Automated Anatomical Labels atlas that corresponded to the overlapping regions were selected for the final mask.

Supplementary Table 1: Peak activations of the Self-Initiative task for the *free*, *timed choice*, and *no choice* conditions, all significant $p < .05$ FWE cluster-corrected (initial threshold $p < .001$, uncorrected).

Main task effect (N=39)	Region (AAL)	BA	Cluster size (voxels)	Side	T-value	*p*-value (FWE)	MNI Coordinates		
							x	y	z
Free	Supramarginal gyrus	40	989	R	7.23	<.001	63	-43	41
	Inferior parietal lobule	40		R	6.68	<.001	57	-34	50
	Supramarginal gyrus	40		R	6.34	<.001	63	-40	26
	Middle frontal gyrus	46	3671	R	7.15	<.001	27	44	32
	Inferior frontal gyrus	44		R	6.91	<.001	51	14	5
	Supplementary motor area	6		R	6.79	<.001	9	14	50
	Cerebellum crus 1	-	426	L	6.13	<.001	-45	-58	-34
	Lingual gyrus	19		L	4.72	<.001	-21	-79	-10
	Cerebellum lobule 6	-		L	3.78	<.001	-27	-64	-16
	Precentral gyrus	6	982	L	5.97	<.001	-60	8	26
	Middle frontal gyrus	46		L	5.80	<.001	-30	50	23
	Middle frontal gyrus	46		L	5.02	<.001	-24	53	7
	Cerebellum crus 1	-	543	R	5.02	<.001	45	-58	-34
	Cerebellum crus 1	-		R	4.98	<.001	33	-52	-34
	Lingual gyrus	19		R	4.37	<.001	33	-70	-16
	Superior occipital gyrus	18	104	R	4.74	<.001	30	-94	11
	Superior occipital gyrus	18		R	4.69	<.001	18	-97	8
	Precuneus	7	91	R	4.91	<.001	12	-58	53
	Precuneus	7		R	3.91	.001	21	-70	53
Timed Choice	Middle occipital gyrus	19	11999	L	10.4	<.001	-48	-79	-1
	Middle occipital gyrus	19		R	9.70	<.001	45	-76	-7
	Cerebellum	-		R	9.66	<.001	15	-73	-19

	Insula	-	457	L	6.70	<.001	-45	11	- 1
	Precentral gyrus	6		L	5.07	<.001	-57	8	26
	Insula	-		L	5.02	<.001	-36	- 4	14
	Midbrain	-	93	R	6.56	<.001	9	-22	-13
	Midbrain	-		L	4.41	<.001	-12	-16	-13
	Hippocampus	-		R	3.98	<.001	24	-25	- 7
	Middle frontal gyrus	45	353	R	4.59	<.001	45	41	11
	Middle frontal gyrus	46		R	4.56	<.001	39	56	- 4
	Inferior frontal gyrus	45		R	4.53	<.001	51	41	- 4
	Medial frontal gyrus	10	129	R	5.49	.012	6	59	-10
No Choice	Superior occipital gyrus	18	4038	R	8.88	<.001	21	-88	14
	Inferior occipital gyrus	19		R	8.83	<.001	36	-70	-10
	Cerebellum lobule 6	-		R	8.69	<.001	27	-58	-22
	Insula		169	L	7.96	<.001	-48	- 1	11
	Inferior frontal gyrus (opercular part)			L	4.07	<.001	-60	8	8
	Cerebellum lobule 8	-	87	R	7.19	<.001	21	-61	-49
	Precentral gyrus	6	734	L	6.70	<.001	-33	-19	68
	Inferior parietal lobule	40		L	6.59	<.001	-45	-28	44
	Supramarginal gyrus	40		L	6.54	<.001	-54	-25	23

AAL= Automated Anatomic Labeling; BA = Brodmann area; FWE = Family-Wise Error corrected, on cluster level; MNI = Montreal Neurological Institute

Supplementary Table 2: Uncorrected peak activations of the of the Self-Initiative task ROI-analyses for the *free*, *timed choice*, and *no choice* conditions, for the total group (upper panel) and high versus low apathy groups (lower panel)

Effects of apathy	Region (AAL)	BA	Cluster size (voxels)	Side	T-value	p-value (<.001, unc)	MNI coordinates x	y	z
Free									
Positive	Angular_L (65)	7	1	L	3.36	.001	-33	-58	38
Negative	No suprathreshold voxels at p<.001 uncorrected								
Choice									
Positive	Frontal_Sup_L (3)	9	4	L	3.69	<.001	-24	41	47
Negative	No suprathreshold voxels at p<.001 uncorrected								
No choice									
Positive	No suprathreshold voxels at p<.001 uncorrected								
Negative	Rolandic_Oper_L (17)	6	3	L	3.94	<.001	-48	5	16
Free									
Low < high	No suprathreshold voxels at p<.001 uncorrected								
Low > high	No suprathreshold voxels at p<.001 uncorrected								
Choice									
Low < high	Frontal_Sup_L (3)	9	2	L	3.35	.001	-12	47	47
Low > high	Frontal_Inf_Oper_R (12)	48	26	R	4.82	<.001	48	11	5
	SupraMarginal_R (64)	48	3	R	3.68	<.001	45	-37	29
	Rolandic_Oper_R (18)	48	5	R	3.68	<.001	57	11	23
	Precentral_L (1)	6	1	L	3.38	.001	-57	5	26
	Precentral_L (1)	6	1	L	3.35	.001	-60	8	29
No choice									
Low < high	No suprathreshold voxels at p<.001 uncorrected								
Low > high	ParaHippocampal_R (40)	37	1	R	3.33	.001	27	-31	-10

AAL= Automated Anatomic Labelling; BA = Brodmann area; FWE = Family-Wise Error corrected, on cluster level; MNI = Montreal Neurological Institute

Supplementary Table 3: Response times and response types per condition, and associations with apathy (as measured with the AES-S).

Responses per condition		Total group Mean (SD) (N=39)*	Low apathy Mean (SD) (N=20)ˣ	High apathy Mean (SD) (N=19)ˣ
Free				
	Response times[1]	2.89 (2.58)	2.38 (2.31)	3.43 (2.8)
	Left button presses[2]	47.24 (5.16)	47.38 (5.38)	47.09 (5.07)
	Variability[3]	59.54 (9.91)	57.69 (7.47)	61.48 (11.85)
Timed Choice				
	Response times[1]	.41 (.11)	.37 (.06)	.44 (.14)
	Left button presses[2]	44.23 (10.11)	44.83 (9.66)	43.6 (10.79)
	Variability[3]	58.79 (15.04)	57.94 (14.56)	59.69 (15.88)
No choice				
	Response times[1]	.43 (.07)	.42 (.05)	.45 (.08)
	Accuracy[4]	96.9 (3.14)	97.56 (2.74)	96.21 (3.45)

*Correlations between group means and AES-S were calculated with a Kendall's Tau Test (τ)± all non-significant

ˣ Two samples T-tests were performed to evaluate group differences: all non-significant

[1] mean RT in s

[2] proportion of left button presses in percentages

[3] variability of response types in percentages

[4] accuracy of response in percentages

Supplementary Table 4: Classification accuracies to differentiate the two apathy groups, displayed per condition

Property/Condition	Free		Choice		No choice	
Total accuracy	54%		59%		44%	
Balanced accuracy	54%		59%		44%	
BA p-value	.28		.11		.68	
Class accuracy (low and high)	50%	58%	50%	68%	45%	42%
CA p-value	.58	0.27	.59	.06	.71	.68
Class predictive value (low and high)	56%	52%	63%	57%	45%	42%

BA = balanced accuracy; CA = Class accuracy
